# Surgery versus conservative drug therapy in alveolar echinococcosis patients in Germany – A health-related quality of life comparison

**DOI:** 10.1016/j.fawpar.2019.e00057

**Published:** 2019-05-30

**Authors:** Julian Schmidberger, Julia Steinbach, Patrycja Schlingeloff, Wolfgang Kratzer, Beate Grüner

**Affiliations:** Department of Internal Medicine I, Ulm University Hospital, Albert-Einstein-Allee 23, 89081 Ulm, Germany; Department of Internal Medicine III, Ulm University Hospital, Albert-Einstein-Allee 23, 89081 Ulm, Germany

**Keywords:** Alveolar echinococcosis, *Echinococcus multilocularis*, SF-36, Therapy

## Abstract

Alveolar echinococcosis (AE) is a rare zoonosis caused by the parasite *Echinococcus multilocularis*. Nothing is known about the health-related quality of life (HRQoL) in patients with AE receiving different types of therapy. Therefore, the aim of the study was to compare HRQoL in patients with AE in Germany depending on their therapeutic regimen namely conservative drug treatment with long-term benzimidazoles versus surgical therapy by resection of the parasitic liver lesions. The 36-Item Short Form Health Survey (SF-36) questionnaire, including other echinococcosis-related questions, was used to measure HRQoL. The SF-36 scales were evaluated according to the algorithms provided by the authors. The statistical analysis was performed with SAS version 9.2. The significance level was set at α = 0.05, *p* < 0.05 corresponds to statistical significance. The investigated group consisted of conservatively drug treated (*n* = 30) and patients with curative surgery (*n* = 25) with confirmed AE. The study was performed at an infectious disease outpatient department from April 2018 to October 2018. The conservatively drug treated patient group consisted of 15 men (50.0%) and 15 women (50.0%) with an average age of 55.7 ± 16.7 years (Median: 59). The surgery group consisted of nine men (36.0%) and 16 women (64.3%) with an average age of 53.3 ± 31.9 years (Median: 54). The physical quality of life of the conservatively drug treated patients did not show any significant differences to the surgical treated group (45.2 ± 11.4 vs. 47.6 ± 9.9; *p* = 0.4079). There was also no significant difference regarding the mental quality of life between the conservatively drug treated patients, and those treated with curative surgery (45.5 ± 10.6 vs. 47.3 ± 10.8; 0.5206). Nevertheless, there was a slight advantage in the physical and mental scores of the patients treated with surgery. Furthermore, for 13 of the 25 surgically treated patients, some aspects of the HRQoL improved significantly after surgery.

The evaluation showed no statistically significant differences in HRQoL in patients with AE dependent on the applied treatment strategy (conservative drug versus curative surgical therapy).

## Highlights

• Health-related quality of life in alveolar echinococcosis patients was analyzed.
• Two treatment groups were compared: conservative drug treatment vs. curative surgery.
• No differences in physical quality of life were found between the groups.
• There was no difference in mental quality of life between the groups.
• Quality of life increased significantly in 52% of surgery patients after treatment.

## 1 Introduction

Alveolar echinococcosis (AE) is a rare but dangerous zoonosis caused by the larval stage *Echinococcus multilocularis. E. multilocularis* is predominantly found in the temperate to cold latitudes of the Northern hemisphere (Kern et al., 2003; Deplazes et al., 2017). In some areas of Southern Germany, Eastern France, Northern Switzerland and Western Austria, more and more cases of disease and pathogen detection are being registered, possibly also through better diagnostic possibilities (Kern et al., 2003; Torgerson et al., 2010; Deplazes et al., 2017). Outside Central Europe human cases are reported from China with the Tibetan plateau and Mongolia, Northern Japan, Russia with Siberia, parts of Turkey, as well as parts of Canada and Alaska (Craig et al., 1992; Craig, 2006; Zhang et al., 2015; Deplazes et al., 2017). AE acts like a malignancy and predominantly affects the liver in >98% of cases but can grow infiltrating to adjacent structures and organs and also has the potential to produce distant ‘metastases’. Classifications for ultrasound and computer tomography help to classify and diagnose the different morphological manifestations of AE in a standardized way (Kratzer et al., 2015; Graeter et al., 2016).

For AE, an indication for therapy is given due to the malignant behavior of the disease and to limit the spread in the organism (Reuter et al., 2002; Vuitton, 2009; Hillenbrand et al., 2017). Depending on the individual patient situation, drug or surgical therapy is available, taking into account the patient-specific stage of the disease. Treatment and prognosis of patients with AE has improved significantly in recent decades, also due to better imaging and immunohistological methods, operational techniques and therapeutic procedures (Vuitton, 2009). The complete resection of the entire parasitic lesion is the goal of surgical therapy (Buttenschoen et al., 2009). In Germany, only 1/3 of patients are operable at the time of diagnosis with a curative resection rate of 42% in Germany (Buttenschoen et al., 2009; Hillenbrand 2017). In other countries, significantly higher rates of curative resection are reported (Joliat et al., 2015).

Unfortunately, most patients are diagnosed with advanced AE, which often means local inoperability. Drug therapy is indicated for all patients diagnosed with AE and carried out with benzimidazoles (BMZ), mostly albendazole (ABZ), less commonly mebendazole (MBZ) (Reuter et al., 2002; Vuitton, 2009), both used as a long-term treatment (Lundström-Stadelmann et al., 2019). With the introduction of benzimidazoles into therapy in 1976, the prognosis improved considerably (Reuter et al., 2002; Vuitton, 2009). If left untreated, the lethality of AE is reported to be 90% within 10–15 years after diagnosis (Chen et al., 2018).

Generally, the life-expectancy of benzimidazole-treated AE patients is not significantly reduced, if therapy is well tolerated. ABZ is given at a dose of 10–15 mg/kg/day in 2 divided doses, taken with a high-fat meal to ensure absorption (Brunetti et al., 2010). The most common side effect is an increase in serum alanine aminotransferases (Vuitton and Bresson-Hadni, 2013). Other important side effects are leukopenia and thrombocytopenia. In addition, alopecia, dizziness, headache, fever, rash, urticaria and anaphylaxis are found in <3% of patients and minor side effects such as abdominal pain, dyspepsia, nausea and vomiting in 4–11% of patients (WHO, 1996). Patients repeatedly complain about the stressful factors of BMZ therapy as well as echinococcosis-associated complaints and psychological stress as part of their care and monitoring. First studies show that the quality of life in patients with AE is reduced compared to the healthy population (Schmidberger et al., 2019; Nikendei et al., 2019).

In medicine, health science, and epidemiological research, the term quality of life describes a multidimensional subjective construct of social, physical and psychological components and includes both positive and negative aspects of individual perception, which often makes it difficult to define the term precisely. The World Health Organization (WHO) describes quality of life as the physical, mental and social well-being of the individual according to the definition of health (Bullinger and Kirchberger, 1998; Bullinger, 2014; Ellert and Kurth, 2013). In the Bangkok Charter for Health Promotion in a Globalized World, drawn up in 2005, the WHO recognizes quality of life as the overriding goal of health promotion (WHO, 2005).

The original focus in medicine was to measure the epidemiological relevance of a disease to a population by determining mortality, but this approach has altered greatly in recent years. Demographic shifts, increasing life expectancy, and improved treatment outcomes create more chronic diseases and thus determine the spectrum of morbidity. Quality of life as an indicator of health has become more relevant and will probably continue to play an increasingly important role. The medical origins of quality of life research can be traced back to oncology and palliative care (Bullinger and Kirchberger, 1998; Bullinger, 2014). In recent years, many different disease-spanning and disease-specific methods for measuring quality of life have been developed from quality of life research (Bullinger, 2014). Especially in oncology, palliative medicine, and allergology, quality of life research plays a central role and is increasingly applied in rehabilitation medicine, epidemiological research, and public health for population-specific investigations (Bullinger and Kirchberger, 1998; Bullinger, 2014). The areas of application of this cross-disease instrument are very diverse and are used both, for sick people and for healthy people aged 14 and over. In the American region, it is frequently used in somatic medicine as well as for people with mental illnesses (Bullinger and Kirchberger, 1998; Bullinger, 2014). The areas of application in Germany range from oncology and palliative medicine to orthopaedic and rehabilitation research. Quality of life is also increasingly being used to assess the quality of different forms of treatment (Bullinger, 2014; Schmidberger et al., 2019).

In the present study, we examined and evaluated whether the HRQoL in patients with AE in Germany differed physically as well as mentally with regard to treatment regimen. In particular, AE patients treated long-term with benzimidazoles as a conservative drug treatment were compared with AE patients treated with curative surgical resection of parasitic liver lesion.

## 2 Materials and methods

### 2.1 Inclusion and exclusion criteria

From the national echinococcosis registry in Germany, 66 patients aged 17–81 years were prospectively enrolled. From this study group 55 patients took part in the survey, 11 patients did not take part in the survey. This corresponds to a representative response of 83%. The study collective was divided into two subgroups; one group consisted of 30 conservatively drug-treated patients (with long-term benzimidazoles), the other group of 25 patients with surgical R0-resection in curative intention. The patients included in the surgical group were treated after surgery with BMZ in accordance with the IWGE guidelines (Brunetti et al., 2010). The study on the HRQoL of patients with AE was conducted at an infectious disease outpatient department in the period from April 2018 to September 2018. Patients in whom AE has so far only been considered possible according to the WHO case definition and patients with incomplete questionnaires were excluded from the study (Brunetti et al., 2010). The study was approved by the local Ethics Committee approval and the Declaration of Helsinki (ref. No. 63/18).

### 2.2 Questionnaire SF-36

The investigation of the HRQoL in patients with AE depending on different forms of therapy was carried out using the 36-Item Short Form Health Survey (SF-36) questionnaire. The SF-36 questionnaire is a worldwide non disease-specific questionnaire to measure HRQoL. It was developed in the context of the Medical Outcome Study (MOS) by the Research and Development Corporation (RAND) (Bullinger and Kirchberger, 1998; Bullinger, 2014). The SF-36 questionnaire consists of 36 items, which form 8 subjective health dimensions and can be summarized to a physical and mental sum scale and are usually referred to as physical and mental quality of life. The dimensions of the SF-36 have a range of 0–100, where 0 represents the worst possible condition and 100 the best possible condition (Bullinger, 2014). The 36 questions of the questionnaire form the physical and mental sum scale according to the evaluation algorithms provided by the authors. The physical quality of life, as well as the mental quality of life, is composed of four different scales of subjective health. The total score for the physical quality of life comprises the scales of ‘physical functioning’, ‘role limitations due to physical health’, ‘bodily pain’ and ‘general health’. The overall score for mental quality of life is composed of the scales ‘emotional well-being’, ‘role limitation due to emotional problems’, ‘social functioning’ and ‘energy/fatigue’. The SF-36 questionnaire was supplemented by further echinococcosis-associated questions on surgery and BMZ therapy.

### 2.3 Statistical analysis

The evaluation was carried out with the statistical software SAS Version 9.2. The scales of the SF-36 questionnaire were formed according to the algorithms provided by the authors (Bullinger, 2014). A simple variance analysis (ANOVA) was used for the hypothesis test and the multivariate covariance analysis (MANCOVA) was used for the determination of possible confounding variables. The null hypothesis (H0) was the assumption that the AE patients showed no differences in quality of life regarding conservative drug treatment with long-term benzimidazoles versus surgical therapy in Germany. The alternative hypothesis (H1) assumed that the AE cases showed a difference regarding conservative drug treatment with long-term benzimidazoles versus surgical therapy in Germany. The level of significance was set at α = 0.05, p < 0.05 corresponds to statistical significance.

## 3 Results

The evaluable collective consisted of *n* = 30 (54.5%) patients with conservative drug therapy and *n* = 25 (45.5%) patients with curative surgery with confirmed AE. The conservatively drug treated patient group consisted of 15 men (50.0%) and 15 women (50.0%) with an average age of 55.7 ± 16.7 years (Median: 59). At the time of data collection, 14 patients (46.7%) were <55 years, 13 patients (43.3%) between 55 and 75 years, and three patients (10.0%) > 75 years (Table 1). The curative surgery group consisted of nine (36.0%) men and 16 (64.3%) women with an average age of 53.3 ± 31.9 years (Median: 54). At the time of data collection, 13 patients (52.0%) were < 55 years old, ten patients (40.0%) between 55 and 75 years old, and two (8.0%) of the patients >75 years old (Table 1).

**Table 1:** Patient characteristics of the conservatively (*n* = 30) and surgically treated patients (*n* = 25).

n	Conservative drug therapy (n = 30)	Curative surgery (n = 25)
	N (%)	N (%)
Gender		
Male	15 (50.0%)	9 (36.0%)
Female	15 (50.0%)	16 (64.3%)
Age		
<55 years	14 (46.7%)	13 (52.0%)
55–75 years	13 (43.3%)	10 (40.0%)
>75 years	3 (10.0%)	2 (8.0%)
Body mass index (BMI)		
BMI < 25	19 (63.3%)	14 (56.0%)
BMI 25–30	8 (26.7%)	9 (36.0%)
BMI > 30	3 (10.0%)	2 (8.0%)
Surgical technique		
Hemihepatectomy		14 (56.0%)
Partial liver resection		11 (44.0%)
Postoperative BMZ therapy		
BMZ 2 years		14 (56.0%)
BMZ < 2 years		9 (36.0%)
BMZ > 2 years		2 (8.0%)


	M ± SD Median	Min-Max	M ± SD Median	Min-Max
Age (years)	55.7 ± 16.7 59.0	17–81	53.3 ± 13.9 54.0	32–79
Body-Mass-Index (BMI)	24.6 ± 3.7 24	18.0–33.4	24.7 ± 3.5 24.6	19.6–31.9
Duration of disease (in months)	120.3 ± 117.0 65.5	4–385	105.0 ± 63.7 89.0	24–265
Duration from diagnosis to surgery (days)	–	–	21.7 ± 50.1 3.0	0–184

*M* *=* *mean; SD* *=* *standard deviation; BMI* *=* *body mass index; n* *=* *sample size.*

In the group of conservatively drug treated patients, the mean BMI was 24.6 ± 3.7. 19 patients (63.3%) in this group had a BMI of <25, eight patients (26.7%) had a BMI of between 25 and 30 and three patients (10.0%) had a BMI of >30. Comparable values were found in the group of patients receiving curative surgery. In this group the mean BMI was 24.7 ± 3.5. In 14 patients (56.0%) of this group the BMI was <25, in nine patients (36.0%) the BMI was between 25 and 30 and two of the patients (8.0%) had a BMI of >30. At the time of data collection, the mean duration of disease in the group of conservatively drug treated patients was 120.3 ± 117.0 months (4 minimum–385 maximum) (Table 1). The average duration of disease of the surgically treated patients (*n* = 25) was 105.0 ± 63.7 months (24 minimum −265 maximum). The time from initial diagnosis to surgery was 21.7 ± 50.1 months (0 minimum – 184 maximum). Of the 25 patients who underwent curative surgery, 14 (56.0%) received a hemihepatectomy and 11 (44.0%) a partial liver resection. Of the surgically treated patients, 14 (56.0%) were treated with BMZ for 2 years postoperatively according to the IWGE guidelines (Brunetti et al., 2010). A total of 9 (36.0%) patients received a longer postoperative BMZ therapy. In 2 (8.0%) patients, the duration of postoperative BMZ therapy was <2 years due to BMZ intolerance and toxicity that was leading to treatment discontinuation (Table 1). In the group of conservatively drug treated patients there was no BMZ intolerance or toxicity leading to treatment discontinuation.

### 3.1 Evaluation of the SF-36 scales

**Fig. 1:**
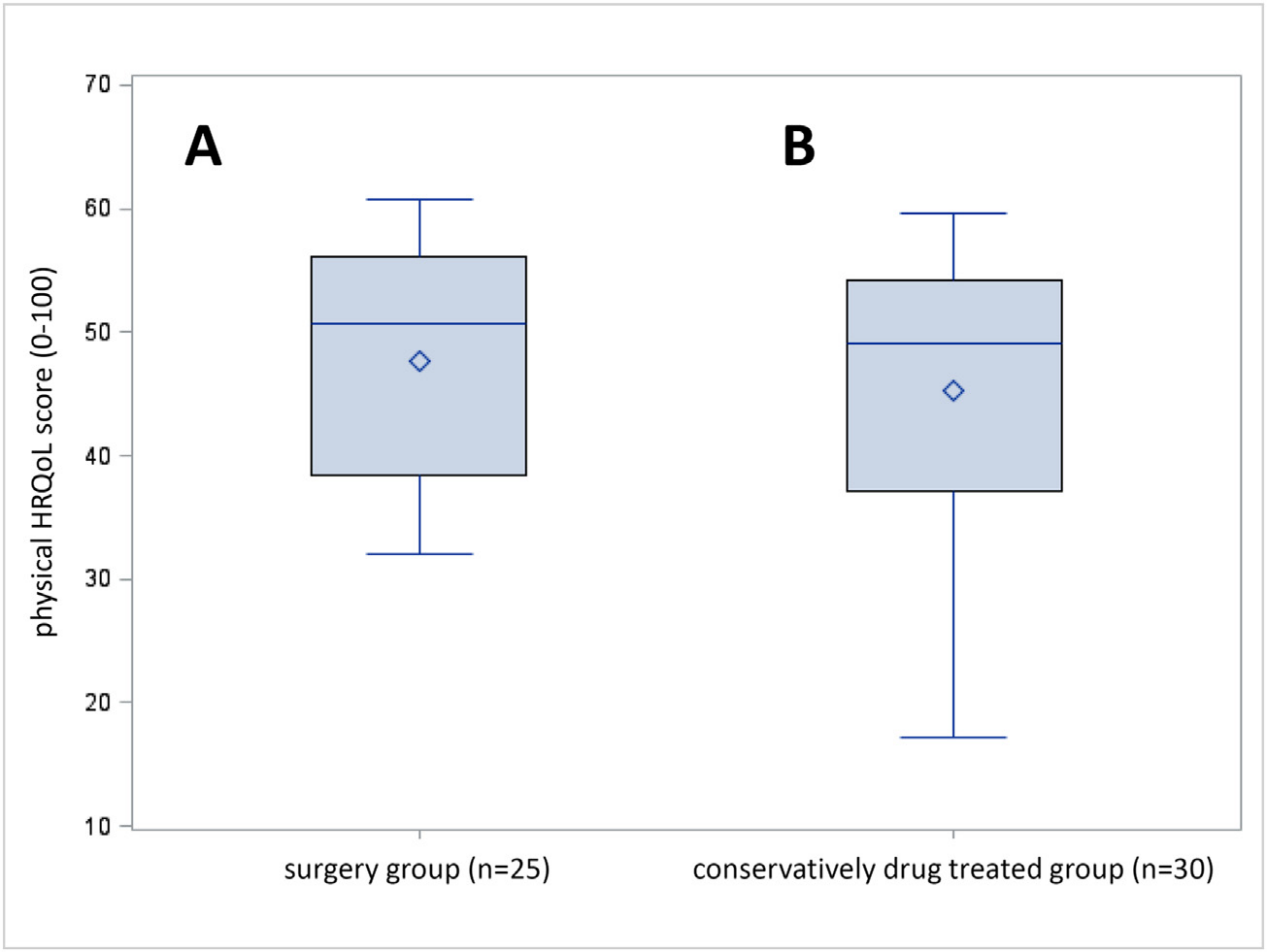
Boxplot representation of the physical sum scale after surgical (A) and drug therapy (B).

**Fig. 2:**
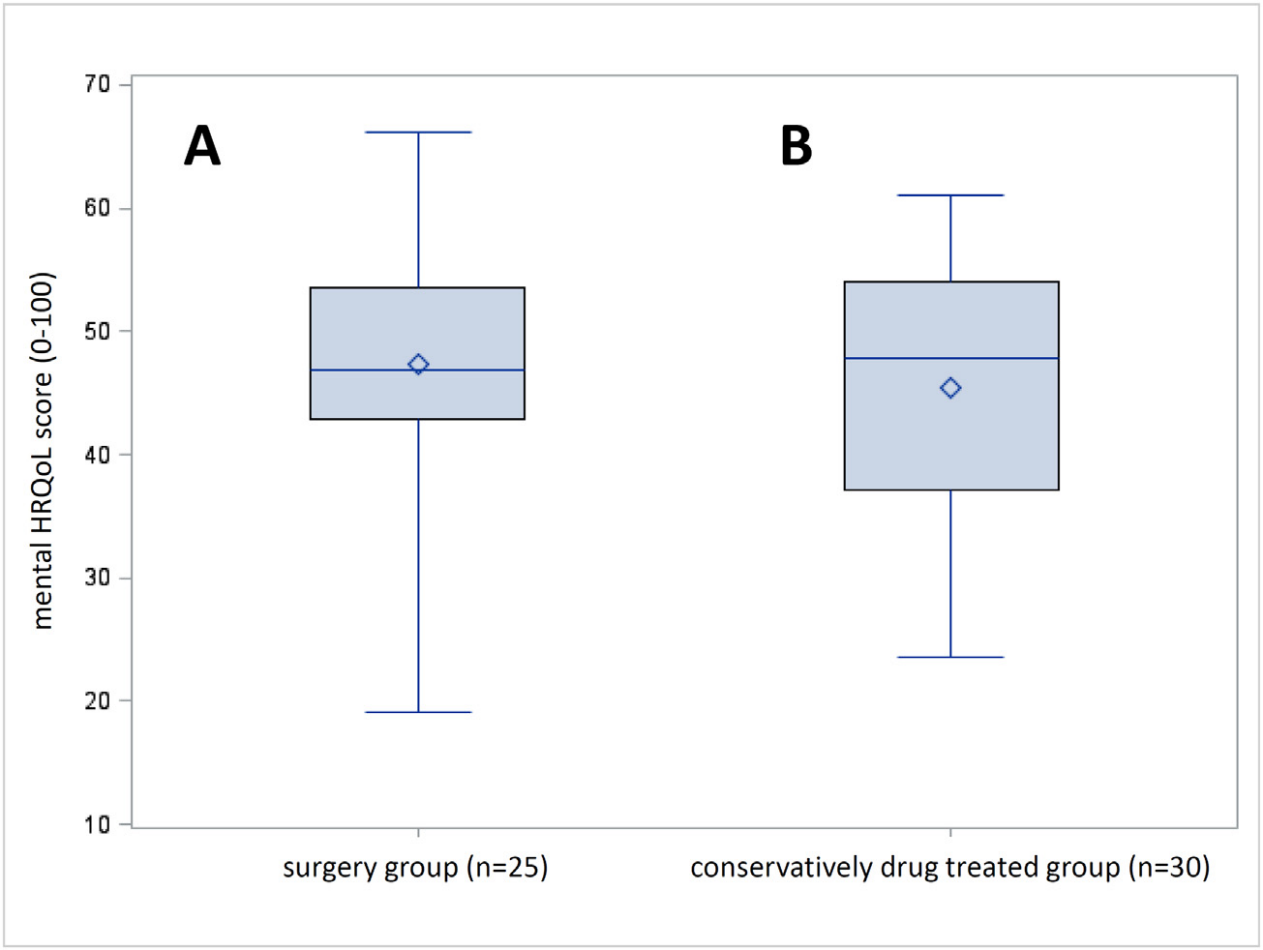
Boxplot representation of the mental sum scale after surgical (A) and drug therapy (B).

The physical quality of life of the conservatively drug treated patients showed no statistically significant differences to the surgery group (45.2 ± 11.4 vs. 47.6 ± 9.9; *p* = 0.4079) (Fig. 1). For the mental quality of life there were also no statistically significant differences between conservatively drug treated patients and those treated with curative surgery (45.5 ± 10.6 vs. 47.3 ± 10.8; 0.5206) (Fig. 2).

#### 3.1.1 Physical quality of life

The scales of ‘physical functioning’ (74.5 ± 24.9 vs. 81.8 ± 21.0; *p*-value = 0.2435), ‘role limitations due to physical health’ (65.0 ± 42.8 vs. 69.0 ± 43.5; p-value = 0.7338), ‘bodily pain’ (72.2 ± 28.4 vs. 73.5 ± 25.5; p-value = 0.9215) and ‘general health’ (51.1 ± 21.4 vs. 61.2 ± 23.5; *p*-value = 0.1042) showed no statistically significant differences between conservatively drug treated patients and those treated with curative surgery (Table 2).

**Table 2:** Mean values and standard deviations (SD) of the 36-Item Short Form Health Survey (SF-36) questionnaire scales in conservatively (n = 30) and surgically treated patients (*n* = 25).

SF scale	Conservative drug therapy (n = 30)	Curative Surgery (n = 25)	*p*-Value
	mean ± SD	mean ± SD	
Physical functioning	74.5 ± 24.9	81.8 ± 21.0	0.2435
Role limitations due to physical health	65.0 ± 42.8	69.0 ± 43.5	0.7338
Bodily pain	72.2 ± 28.4	73.5 ± 25.5	0.9215
General health	51.1 ± 21.4	61.2 ± 23.5	0.1042
Energy/fatigue	49.2 ± 22.7	56.6 ± 18.4	0.1858
Social functioning	72.9 ± 26.5	78.0 ± 22.6	0.4460
Role limitations due to emotional problems	67.8 ± 40.6	73.3 ± 40.8	0.6165
Emotional well-being	67.6 ± 17.9	69.9 ± 19.0	0.6459
Physical summary score	45.2 ± 11.4	47.6 ± 9.9	0.4079
Mental summary score	45.5 ± 10.6	47.3 ± 10.8	0.5206

#### 3.1.2 Mental quality of life

The scales for ‘emotional well-being’ (49.2 ± 22.7 vs. 56.6 ± 18.4; p-value = 0.1858), ‘role limitations due to emotional problems' (72.9 ± 26.5 vs. 78.0 ± 22.6; p-value = 0.4460), ‘social functioning’ (67.8 ± 40.6 vs. 73.3 ± 40.8; p-value = 0.6165) and ‘energy/fatigue’ (67.6 ± 17.9 vs. 69.9 ± 19.0; p-value = 0.6459) also showed no statistically significant differences between conservatively drug treated patients and those treated with curative surgery (Table 2).

### 3.2 Multivariate analysis of variance

The multivariate analysis of variance did not show a statistically significant influence of the possible confounding variables (p-value = 0.3898) on the physical sum scale, taking into account discussable confounding variables such as age (p-value = 0.3570), BMI (p-value = 0.1698), gender (p-value = 0.1113) and initial diagnosis or duration of disease (p-value = 0.1412) (Table 3). The mental quality of life was considered among the same discussable confounding variables such as age (p-value = 0.4381), BMI (p-value = 0.5577), gender (p-value = 0.9197) and initial diagnosis or duration of disease in the multivariate analysis of variance. Again, there was no statistically significant influence of the possible confounding variables (p-value = 0.3522) (Table 3).

**Table 3:** Results of the Analysis of Variance and Multivariate Analysis of Variance (ANOVA and MANOVA) for physical and mental quality of life.

Physical quality of life					
Source	DF	Sum of squares	Mean square	F	Pr > F
Model	7	898.496950	128.356707	1.13	0.3641
Error	44	5013.261078	113.937752		
Corrected total	51	5911.758028			


Physical quality of life					
	Wilks' lambda	Numerator DF	Denominator DF	F	Pr > F
Age	0.98068688	1	44	0.87	0.3570
BMI	0.95760769	1	44	1.95	0.1698
Gender	0.94337076	1	44	2.64	0.1113
First diagnosis	0.95145973	1	44	2.24	0.1412
Age ∗ BMI	0.99487582	1	44	0.23	0.6364
Age ∗ BMI ∗ Gender	0.96518120	1	44	1.59	0.2144
Age ∗ BMI ∗ Gender ∗ First diagnosis	0.98314078	1	44	0.75	0.3898


Mental quality of life					
Source	DF	Sum of squares	Mean square	F	Pr > F
Model	7	637.015659	91.002237	0.77	0.6134
Error	44	5182.930615	117.793878		
Corrected total	51	5819.946275			


Mental quality of life					
	Wilks' lambda	Numerator DF	Denominator DF	F	Pr > F
Age	0.98627221	1	44	0.61	0.4381
BMI	0.99212992	1	44	0.35	0.5577
Gender	0.99976665	1	44	0.01	0.9197
First diagnosis	0.99730669	1	44	0.12	0.7320
Age ∗ BMI	0.97619636	1	44	1.07	0.3060
Age ∗ BMI ∗ Gender	0.99652659	1	44	0.15	0.6972
Age ∗ BMI ∗ Gender ∗ First diagnosis	0.98029910	1	44	0.88	0.3522

*BMI* *=* *body mass index; F = F value; Sig* *=* *p-value; DF* *=* *Degree of freedom~ statistically significant with p* *<* *0.05.*

### 3.3 Echinococcosis associated questions

Further echinococcosis-associated questions were asked in the context of the quality of life survey of the patients with curative surgery (*n* = 25). This survey showed that in 13/25 (52%) of these patients the quality of life improved as a result of the surgery. In addition, 12/25 (48%) of the patients interviewed stated that they occasionally felt anxiety or panic that was not felt before the diagnosis was made. The group of conservatively drug treated patients with long-term benzimidazoles also showed that 15/30 (50%) occasionally felt anxiety or panic that was not felt before diagnosis.

In a subgroup analysis in the surgery group, it was examined whether the quality of life differed between those who indicated that the individual quality of life had changed (13 patients) or and those who observed no change as a result of the curative surgery (12 patients). The evaluation revealed a significant increase in the physical quality of life for patients who reported that their quality of life had changed as a result of surgery compared to those who did not report an improvement after surgery (43.13 ± 8.80 vs. 52.47 ± 8.89; *p* = 0.0149). Within the group of physical quality of life, there were significant differences between ‘bodily pain’ (57.69 ± 19.83 vs. 90.58 ± 19.34; *p* = 0.0003) and ‘role limitations due to physical health’ (51.92 ± 48.37 vs. 87.50 ± 29.19; *p* = 0.0362). There were no significant differences in the overall score of mental quality of life between the two groups (44.31 ± 12.75 vs. 50.61 ± 7.47; *p* = 0.1446). However, the group of ‘social functioning’, a component of mental quality of life, showed significant differences between the groups (56.41 ± 47.88 vs. 91.66 ± 20.71; *p* = 0.0273) (Table 4).

**Table 4:** Mean values and standard deviations (SD) of 36-Item Short Form Health Survey (SF-36) questionnaire scales of surgically treated AE patients (n = 25).

SF scale	mean ± SD		*P*-value
	HRQoL after surgery changed (*n* = 13)	HRQoL after surgery not changed (*n* = 12)	
Physical functioning	76.92 ± 20.46	87.08 ± 21.15	0.2355
Role limitations due to physical health	51.92 ± 48.37	87.50 ± 29.19	0.0362⁎
Bodily pain	57.69 ± 19.83	90.58 ± 19.34	0.0003⁎
General health	52.69 ± 19.77	70.41 ± 24.40	0.0602
Energy/fatigue	51.92 ± 18.08	61.66 ± 18.13	0.1922
Social functioning	68.26 ± 25.82	88.54 ± 12.45	0.0212
Role limitations due to emotional problems	56.41 ± 47.88	91.66 ± 20.71	0.0273⁎
Emotional well-being	66.46 ± 21.45	73.66 ± 16.13	0.3505
Physical summary score	43.13 ± 8.80	52.47 ± 8.89	0.0149⁎
Mental summary score	44.31 ± 12.75	50.61 ± 7.47	0.1446

⁎ Statistically significant with *p* < 0.05.

## 4 Discussion

If left untreated, AE has a high long-term mortality (Chen et al., 2018); therefore, treatment is indicated for this disease. Due the lack of early symptoms, AE is often diagnosed in an advanced stage, meaning surgical treatment by complete resection of the entire parasitic mass is only achievable in the minority of patients in Germany. Improved treatment successes with long-term drug treatment using benzimidazoles ensure a chronification of the disease and thus determine the morbidity spectrum, whereby the quality of life becomes increasingly important as an indicator of health and for measuring outcomes (Bullinger, 2014).

This study investigated whether the quality of life in patients with AE in Germany differed depending on the type of therapy. The study found no significant differences in the physical and mental quality of life in patients with AE as a function of surgery and conservative drug therapy. Nevertheless, there was a positive trend for the surgery group.

In a randomized clinical trial, 46 patients under the Enhanced Recovery Program with open liver resection were compared with 45 patients under the standard care group. A significantly better HRQoL and superiority for the surgery group was found (Jones et al., 2013). It should be noted that the patients included were predominantly symptomatic patients with complaints and pain. In contrast to the study by Jones et al., in the case of the present patients with hepatic AE there were often no symptoms and complaints. In particular, the question arises as to whether people with severe complaints often benefit from surgery, whereas cases in which the disease is not associated with severe physical complaints benefit less from an operative therapy.

The present study further showed that in 13/25 (52%) of surgically treated patients the quality of life improved as a result of the curative surgery, in particular for ‘bodily pain’ and ‘physical functioning‘. The HRQoL scores were clearly superior to the group of patients who indicated that HRQoL did not change as a result of the curative surgery. Our data seem to indicate that some symptom-free patients with AE benefit less from surgery and removal of the parasitic lesion than those who are in bodily pain and/or experience impaired physical functioning. However, surgical treatment of AE in curative intention is only to achieve when AE is diagnosed early enough with the opportunity of complete local resection of the parasitic lesion. Potential negative aspects of surgery include postoperative scarring or wound healing disorders caused by the resection of the frequently very large foci and space requirements as well as performance restrictions. Studies have shown that in the course of liver resections, as frequently used in patients with hepatic AE, surgical pain and associated physical limitations can frequently occur after surgery (Bruns et al., 2010). In the study conducted by Bruns et al., only about 50% of the 128 patients examined were pain-free at the time of the survey (Bruns et al., 2010). The study further showed that the removal of large foci is associated with a poorer quality of life compared to the removal of smaller foci (Bruns et al., 2010; Martin et al., 2007).

Our study also showed that 48% of patients in the curative surgery group and 50% in the conservative drug treated patient group occasionally experienced anxiety or panic that was not experienced prior to diagnosis of the parasitic disease. These results show the importance and dimension of mental stress on health and well-being due to parasitic diseases.

A recent study of our group showed that especially mental quality of life is significantly reduced in people with AE compared to the healthy population (Schmidberger et al., 2019). This offers starting points for possible psychotherapeutic measures to improve the HRQoL sustainably and long term. According to our study somatic and preventive measures may also contribute to improving the quality of life in patients with AE. Unfortunately, there are no studies available on this subject in patients with AE, due to the rareness of the disease. Another critical point for those patients suffering from a rare disease, for which fundamental knowledge is lacking among the most primary care physicians, is the feeling of carrying a parasite inside their body. Taking this into account the impaired mental quality of life seems a logic consequence and beside offering psychotherapy and self-help groups the improvement of knowledge about the disease and treatment opportunities might be advantageous for the clinical course of AE patients.

A study on the effects of psychotherapeutic interventions on the quality of life of people with cancer showed that patients benefit from psychotherapeutic interventions, which in turn can lead to greater empowerment of patients (Kalter et al., 2018). Furthermore, the effects of moderators of exercise on quality of life and physical function in patients with AE, similar to those in cancer patients, could also have a positive effect on quality of life (Buffart et al., 2017). Studies with dialysis patients with terminal kidney failure also showed that patients might benefit from a membership in a self-help group (Schmidberger, 2016).

The small sample size of the AE patients examined must clearly be regarded as a limitation of the present study. Nevertheless, the study provides an important overview of the health situation of patients with AE in different forms of therapy in Germany. It should be noted that the study describes the situation in Germany and the outcomes of similar studies in other countries may differ from our results. In the present study, socioeconomic and family factors have not been evaluated so far. These possible confounding factors must be the subject of further research activities. Additionally, further studies with larger case numbers are necessary to confirm the results.

There were no differences in the physical and mental quality of life between patients undergoing curative surgery and patients undergoing conservative drug therapy with long-term benzimidazoles in Germany. Nevertheless, the evaluation showed a slight trend towards better scores in the group of surgically curative treated patients, and 52% of the latter group experienced an improvement in aspects of the HRQoL after surgery. The results of the study may help in the treatment planning of patients with AE in Germany. Depending on the patient situation, an individual therapy decision should be made.
